# Effects of Nitrogen Deficiency on the Photosynthesis, Chlorophyll *a* Fluorescence, Antioxidant System, and Sulfur Compounds in *Oryza sativa*

**DOI:** 10.3390/ijms251910409

**Published:** 2024-09-27

**Authors:** Ling-Hua Chen, Ming Xu, Zuxin Cheng, Lin-Tong Yang

**Affiliations:** 1 Jinshan College of Fujian Agriculture and Forestry University, Fuzhou 350002, China; 000d060126@fafu.edu.cn; 2 Engineering Technology Research Center of Fujian Special Crop Breeding and Utilization, College of Agriculture, Fujian Agriculture and Forestry University, Fuzhou 350002, China; xmfau@fafu.edu.cn (M.X.); 000q010089@fafu.edu.cn (Z.C.); 3 College of Resources and Environment, Fujian Agriculture and Forestry University, Fuzhou 350002, China

**Keywords:** *Oryza sativa*, nitrogen deficiency, photosynthesis, antioxidant system, sulfur metabolism

## Abstract

Decreasing nitrogen (N) supply affected the normal growth of *Oryza sativa* (*O. sativa*) seedlings, reducing CO_2_ assimilation, stomatal conductance (gs), the contents of chlorophylls (Chl) and the ratio of Chl *a*/Chl *b*, but increasing the intercellular CO_2_ concentration. Polyphasic chlorophyll *a* fluorescence transient and relative fluorescence parameters (JIP test) results indicated that N deficiency increased F_o_, but decreased the maximum quantum yield of primary photochemistry (F_v_/F_m_) and the maximum of the IP_phase_, implying that N-limiting condition impaired the whole photo electron transport chain from the donor side of photosystem II (PSII) to the end acceptor side of PSI in *O. sativa*. N deficiency enhanced the activities of the antioxidant enzymes, such as ascorbate peroxidase (APX), guaiacol peroxidase (GuPX), dehydro–ascorbate reductase (DHAR), superoxide dismutase (SOD), glutathione peroxidase (GlPX), glutathione reductase (GR), glutathione S-transferase (GST) and *O*-acetylserine (thiol) lyase (OASTL), and the contents of antioxidant compounds including reduced glutathione (GSH), total glutathione (GSH+GSSG) and non-protein thiol compounds in *O. sativa* leaves. In contrast, the enhanced activities of catalase (CAT), DHAR, GR, GST and OASTL, the enhanced ASC–GSH cycle and content of sulfur-containing compounds might provide protective roles against oxidative stress in *O. sativa* roots under N-limiting conditions. Quantitative real-time PCR (qRT-PCR) analysis indicated that 70% of the enzymes have a consistence between the gene expression pattern and the dynamic of enzyme activity in *O. sativa* leaves under different N supplies, whereas only 60% of the enzymes have a consistence in *O. sativa* roots. Our results suggested that the antioxidant system and sulfur metabolism take part in the response of N limiting condition in *O. sativa*, and this response was different between leaves and roots. Future work should focus on the responsive mechanisms underlying the metabolism of sulfur-containing compounds in *O. sativa* under nutrient deficient especially N-limiting conditions.

## 1. Introduction

Nitrogen (N) is one of principal macro–elements for the normal development and fertility of crops [1]. As a necessary component of various bioactive molecules, such as amino acids, proteins, nucleic acids, adenosine triphosphate, vitamins, and chlorophyll (Chl), it constitutes about 2–4% of plant dry weight (DW) [2]. Therefore, N not only plays a key role in miscellaneous biological processes in plants, but also determines the quality and grain yield of cereal crops. Previous statistics indicate that, among the many factors that could determine and increase rice yields, the contribution of chemical N fertilizer is greater than 40% [3]. However, under current agricultural management practices, the average nitrogen use efficiency (NUE) is about 40–50% in the field. The excessive use of N fertilizers causes water and air pollution, resulting in extensive concerns for the environment (e.g., eutrophication and greenhouse effects) and the global economy [4]. Thus, understanding the physiological and molecular mechanisms underlying the adaptive strategy of rice to N-limiting conditions is critical for environmentally friendly and sustainable cultivation. 

Moderate N nutrition can ensure the normal biosynthesis of photosynthetic pigments and enhance the ability of light energy capture and conversion, improving photochemical efficiency in plant leaves [3,5]. Measurements of CO_2_ assimilation and chlorophyll *a* fluorescence transient are widely adopted methods used to monitor plant growth and development, resistance, and the interactions between a plant and its environment [6]. Studies in the literature indicate that N deficiency decreases the use efficiency of photo-radiation and CO_2_ assimilation (photosynthesis rate) in many annual crops such as *Triticum aestivum* [7], *Sorghum bicolor* [8], *Oryza sativa* [2,9], *Zea mays* [10,11], *Ipomoea batatas* [12], *Helianthus annuus* [13], and perennial tree including *Camellia sinensis* [14] and *Citrus* [15], etc. Although a few studies have attributed the decreased CO_2_ assimilation in plants under N-limiting conditions to stomatal conductance [8,16], most of the previous works suggest that a decline in the biochemical capacity for carboxylation and damaged photosystem II (PSII), revealed by the decreased activity of Rubisco and the diminished maximal efficiency of PSII photochemistry (F_v_/F_m_), respectively, are the key constraining factors for photosynthesis in plants under N-limiting conditions. Furthermore, photochemical and non-photochemical quenching can help plants to grow under low-N conditions [16,17]. 

Due to the decreased light energy utilization efficiency, excessive photo energy inevitably increases the production of reactive oxygen species (ROS) and disrupts the equilibrium of ROS. The strong oxidizing ROS, including superoxide anion radicals (O_2_^–^), hydrogen peroxide (H_2_O_2_), and hydroxyl radicals (OH) may then attack biological molecules, such as nucleic acids, amino acids, carbohydrates, and lipids, inducing the lipid peroxidation cellular membrane revealed by the increased content of malondialdehyde (MDA) [18]. In plant cells, the ROS-scavenging systems, including the ascorbate–glutathione (ASC–GSH) cycle, recruit the enzymatic antioxidants (e.g., superoxide dismutase (SOD), catalase (CAT), ascorbate peroxidase (APX), dehydro–ascorbate reductase (DHAR), monodehydro–ascorbate reductase (MDHAR), glutathione reductase (GR), guaiacol peroxidase (GuPX), and glutathione peroxidase (GlPX)), and non-enzymatic antioxidants (e.g., reduced glutathione (GSH), ascorbic acid (Asc), methionine (Met), thioredoxin (TRX), and phytochelatins (PCs)) to counteract oxidative damage in the chloroplast and cytoplasm [19,20]. The observed enhanced antioxidant system has been reported in many plants under N, phosphorus (P), potassium (K), and sulfur (S) stresses [17,21,22,23,24,25,26]. Furthermore, sulfur is the fourth macronutrient for plants, after N, P, and K, and is vital for normal growth and development from multiple aspects. Sulfur is a constituent of the molecule of methionine, cysteine, GSH, vitamins, PCs, chlorophyll, coenzyme A, and *S*-adenosyl-methionine, which collectively scavenge excessive ROS induced by environmental stresses [27]. Cysteine is the first reduced organic sulfur compound and a central biomolecule in S metabolism in plants; it is synthesized by the reaction of S^2−^ and *O*-acetyl serine (OAS) catalyzed by *O*-acetylserine (thiol) lyase (OASTL) [28]. The synthesized cysteine could be further incorporated into S-containing metabolites such as GSH, Met, biotin, coenzyme A, TRX, iron–sulfur-containing proteins (as ferredoxin), adenosine-5′–phosphosulfate, and other non-protein thiol compounds in cells [29]. The increased content of cysteine has been reported in N-starvation tea leaves and *Citrus* roots [30,31]. 

Rice is one of the most important staple foods in the world. N stress in rice can be caused by both N deficiency and N excess. Increasing the application of N fertilizer is the main impetus for the grain yield production of rice [32]. Some details about the physiological and molecular mechanisms regarding N-deficiency responses in *O. sativa* have been investigated [2]. Our previous research showed that N deficiency affected the metabolism of organic acids and N assimilation in *O. sativa* [1]. However, the details about the effects of N deficiency on the donor side of PSII to the end acceptor side of PSI, and antioxidant system, especially the metabolism of S-containing compounds in *O. sativa* leaves and roots, were unveiled. The aim of this study is to dissect the effects of N deficiency on the main apparatus of photosynthetic electron transport (PET) in thylakoid, the different responses of the antioxidant system and the gene expression pattern of related enzymes between the leaves and roots of *O. sativa* seedlings. Our results can be used to further understand the physiological and molecular responses of *O. sativa* seedlings to N-limiting conditions and for the high-quality cultivation of rice and sustainable development of farmlands.

## 2. Results

### 2.1. Effects of N Deficiency on the Growth of Rice Seedlings

**Figure 1:**
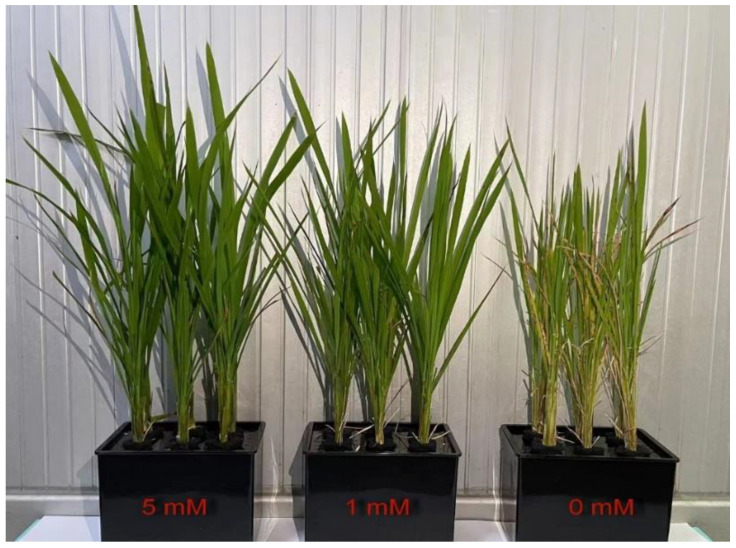
Effects of different nitrogen treatments on the growth of *O. sativa* seedlings. Rice seedlings were transplanted to the Hoagland nutrient solutions containing 5 mM NH_4_NO_3_ (control), 1 mM NH_4_NO_3_ (low N), and 0 mM NH_4_NO_3_ (N deficiency), respectively. Seedlings were cultured under a 14 h light/10 h dark regime with a photo radiation of 150 μmol m^−2^ s^−1^ and a relative humidity of 68% at 28 °C. Three weeks later, rice seedlings were separated into shoots and roots and wrapped with aluminum foil.

Low N and N deficiency apparently inhibited the growth of *O. sativa* seedlings. The height of the seedlings gradually decreased along with the decreasing supply of N. Furthermore, the chlorosis of *O. sativa* leaves was observed in the mature leaves of N-deficient seedlings (Figure 1).

### 2.2. Effects of N Deficiency on Gas Exchange Parameters and Leaf Chlorophylls (Chl) in Rice Seedlings

**Figure 2:**
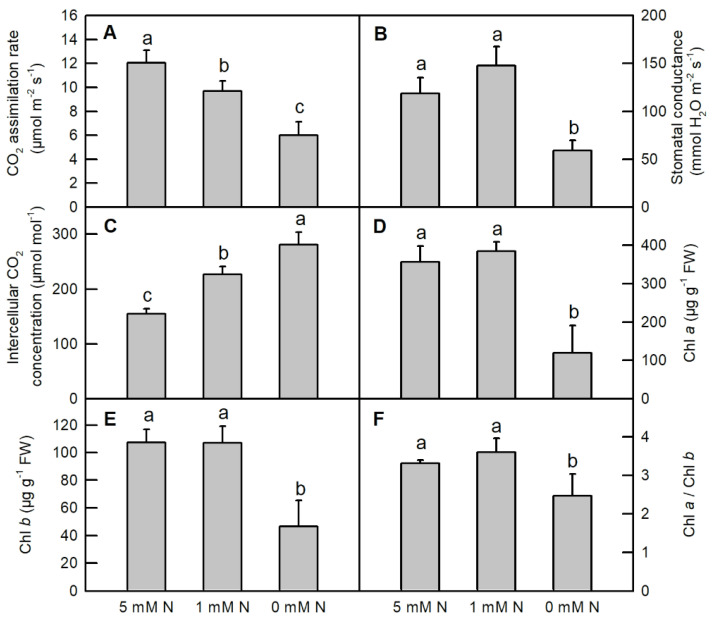
Effects of different nitrogen treatments on CO_2_ assimilation rate (**A**); stomatal conductance ((**B**) *n* = 6); internal CO_2_ concentration ((**C**) *n* = 6); chlorophyll *a* (Chl *a*, (**D**) *n* = 3), Chl *b* ((**E**) *n* = 3) and the ratio of Chl *a* to Chl *b* (Chl *a*/Chl *b* (**F**) *n* = 3) in *O. sativa* seedlings. Bars represent means ± SD (*n* = 6). Different letters above the bars represent a significant difference at *p* < 0.05.

Both low N and N deficiency dramatically decreased the CO_2_ assimilation (decreased by 19.7% and 50%, respectively), whereas these two treatments apparently increased the intercellular CO_2_ concentration in *O. sativa* leaves (increased by 46.7% and 81.9%, respectively) (Figure 2A,C). N deficiency clearly lowered the stomatal conductance (gs), the contents of chlorophyll *a*, chlorophyll *b*, and the ratio of Chl *a*/Chl *b* in *O. sativa* leaves (Figure 2B,D–F). No significant differences in the stomatal conductance, the contents of chlorophyll *a*, chlorophyll *b*, and the ratio of Chl *a*/Chl *b* were observed between low-N leaves and the control ones (Figure 2B,D–F).

### 2.3. Effects of N Deficiency on the Parameters of Chlorophyll a Fluorescence in Rice Leaves

**Figure 3:**
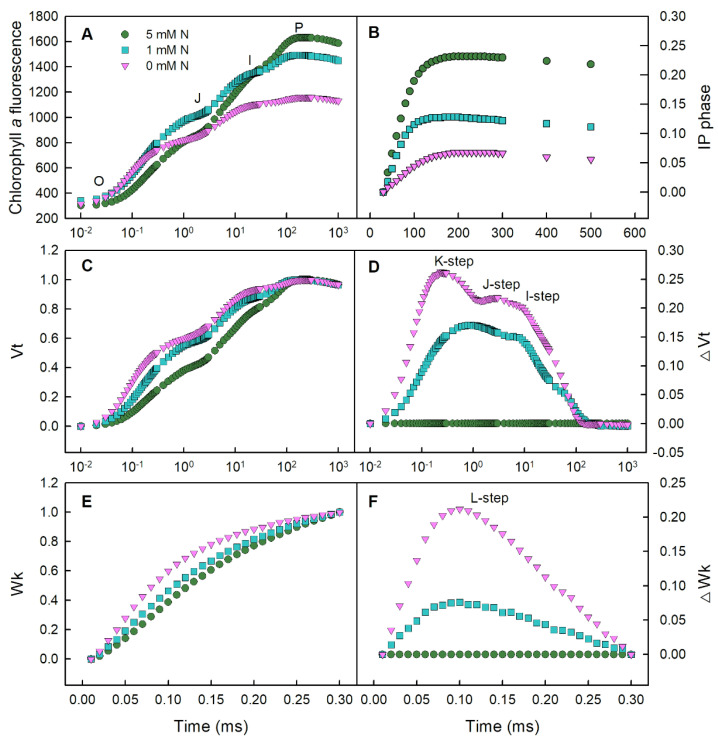
Effects of different nitrogen treatments on the average Chl *a* fluorescence transient (**A**); IP phase: (F_t_ − F_I)_/(F_I_ − F_o_) in dark-adapted *O. sativa* leaves (**B**); the relatively variable fluorescence between F_o_ to F_m_: V_t_ = (F_t_ − F_o_)/(F_m_ − F_o_) (**C**); the differences in V_t_ between low-N and N-deficient samples and the control samples (5 mM N) (**D**); the relatively variable fluorescence between Fo to F_300μs_: W_k_ = (F_t_ − F_o_)/(F_300μs_ − F_o_) (**E**); and the differences in W_k_ between low-N and N-deficient samples and the control samples (5 mM N) (**F**). Values are represented by the means of six biological replicates.

The chlorophyll *a* fluorescence transient (OJIP) showed a typical curve in *O. sativa* leaves under different N treatments (Figure 3A). Increasing N supply in the nutrient solution gradually enhanced the maximum IP phase of *O. sativa* leaves (Figure 3B). The dynamics of relative variable fluorescence (V_t_) and the differences of normalized low-N and N-deficient transients minus the control transient (ΔV_t_) showed that the V_t_ and ΔV_t_ of low-N and N-deficient transients protruded above the control ones (Figure 3C,D). Meanwhile, three positive bands around 300 μs (K-step), 2 ms (J-step) and 30 ms (I-step) were observed in the N-deficient transient, which was more pronounced than the low-N treated samples (Figure 3D). Similarly, a positive band around the immediate vicinity of 120μs (L-step) was observed on the relative variable fluorescence between F_o_ and F_300μs_ (W_K_), and the differences in the normalized low-N and N-deficient transients minus the control transient (ΔW_K_) (Figure 3E,F). 

**Figure 4:**
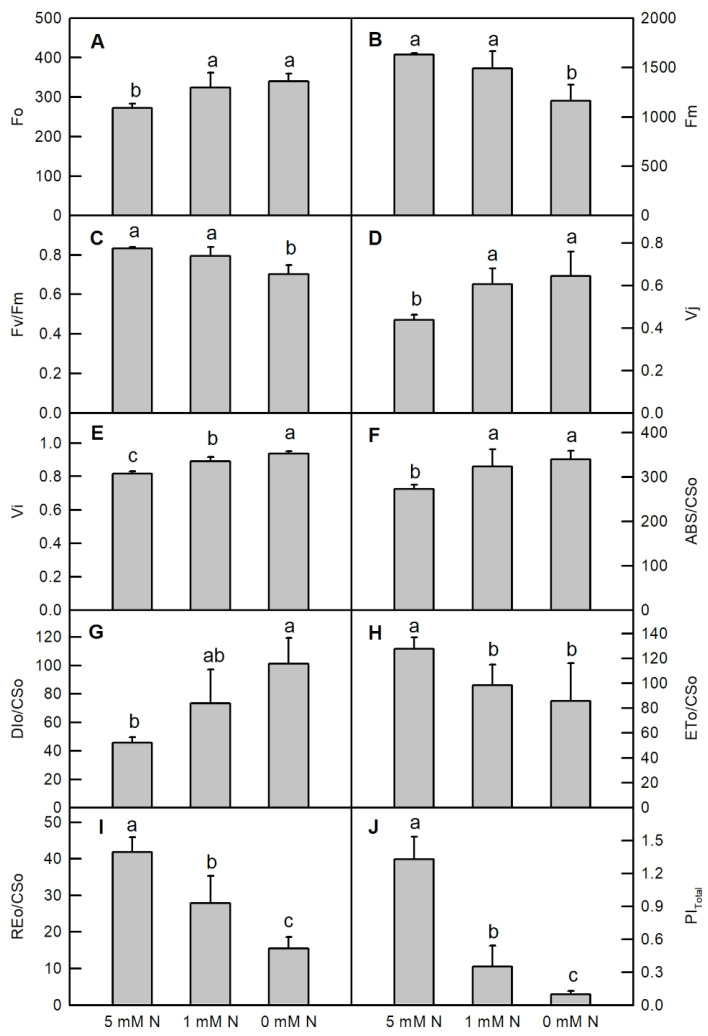
Effects of different nitrogen treatments on the primary fluorescence: F_o_ (**A**); maximum fluorescence: F_m_ (**B**); maximum quantum yield of primary photochemistry: F_m_/F_o_ (**C**); variable fluorescence at t = 2 ms: V_j_ (**D**); variable fluorescence at t = 30 ms: V_i_ (**E**); absorbed photo radiation based on the cross section: ABS/CS_o_ (**F**); dissipated energy based on the cross section: DI_o_/CS_o_ (**G**); energy used in the electron transport: ET_o_/CS_o_ (**H**); energy used in the reduction of end acceptor side of PSI: Re_o_/CS_o_ (**I**); and the total performance index of PSs: PI_total_ (**J**) in dark-adapted *O. sativa* leaves. Bars represent means ± SD (*n* = 6). Different letters above the bars represent a significant difference at *p* < 0.05.

Ten selected primary and calculated fluorescence parameters from different N treated samples are visualized in Figure 4. Compared to the control sample, both low N and N deficiency raised the values of F_o_, V_j_, ABS/CS_o_ and lowered the value of ET_o_ /CS_o_, Re_o_ /CS_o_, and PI_total_ in *O. sativa* leaves (Figure 4A,D,F,H–J). Only the N deficiency treatment significantly decreased the values of F_m_ (decreased by 28.9%), F_v_/F_m_ (decreased by 15.6%), but increased the value of DI_o_/CS_o_ (increased by 128%), when compared to the controls (Figure 4B,C,G). Elevating the N level apparently increased the value of V_i_ in *O. sativa* leaves (Figure 4E). 

### 2.4. Effects of N Deficiency on the Activities of Antioxidant Enzymes in Rice Seedlings

**Figure 5:**
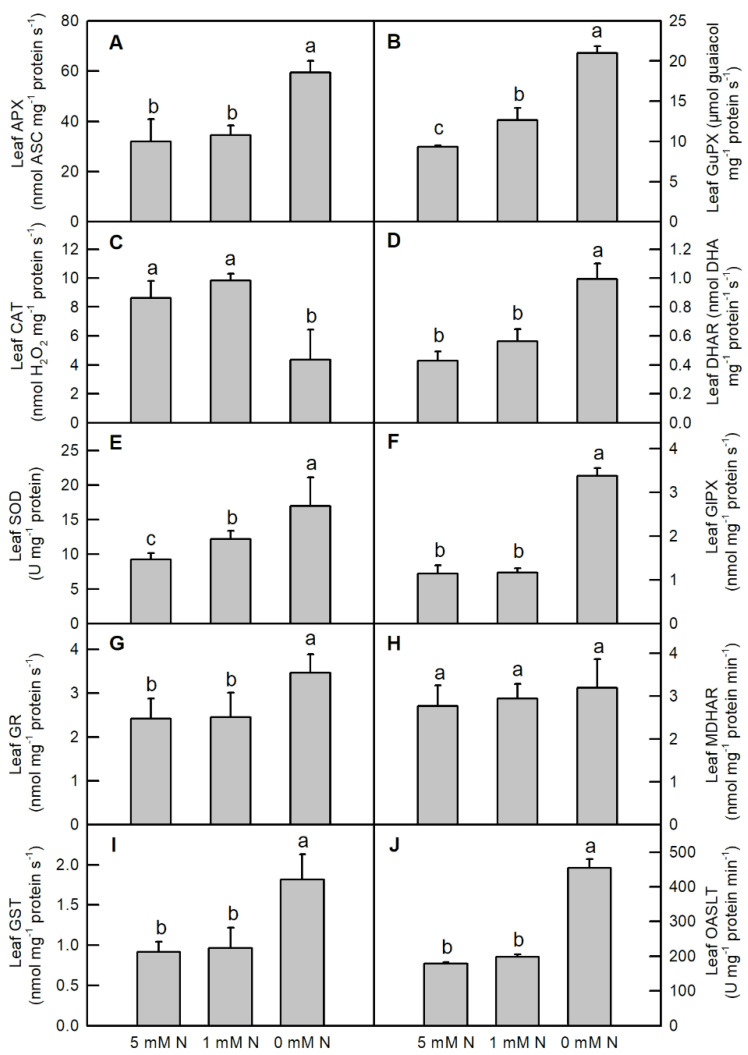
Effects of different nitrogen treatments on the activities of antioxidant enzymes (**A**–**H**); and sulfur metabolism related enzymes (**I**,**J**) in *O. sativa* leaves. Bars represent means ± SD (*n* = 3). Different letters above the bars represent a significant difference at *p* < 0.05.

Nitrogen deficiency dramatically increased the activities of leaf APX (Figure 5A; increased by 86.3%), leaf DHAR (Figure 5D; increased by 132%), leaf GlPX (Figure 5F; increased by 195%), leaf GR (Figure 5G; increased by 43%), and leaf GST (Figure 5I; increased by 98.7%), leaf OASTL (Figure 5J; increased by 155%); however, it lowered the activity of leaf CAT (Figure 5C; decreased by 49.6%) in *O. sativa* seedlings, when compared to the controls. Decreasing the N supply gradually increased the activities of leaf GuPX (Figure 5B) and leaf SOD (Figure 5E), but it did not alter the activity of leaf MDHAR (Figure 5H). Except for increasing the activities of leaf GuPX (Figure 5B) and leaf SOD (Figure 5E), low N did not alter the activities of the abovementioned enzymes in *O. sativa* leaves when compared to the controls. 

**Figure 6:**
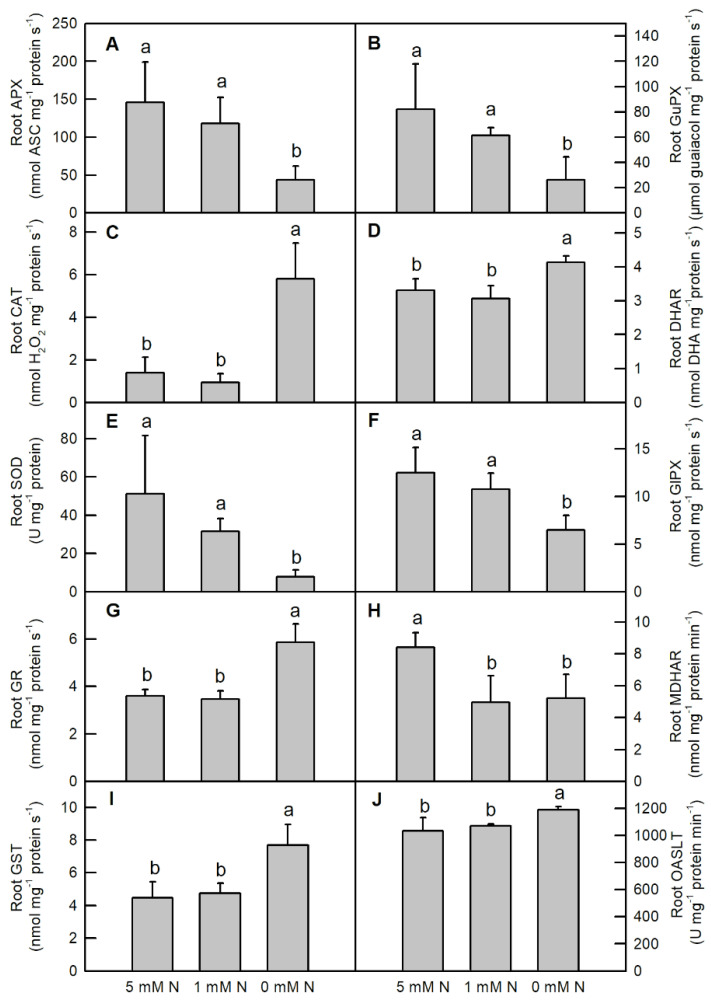
Effects of different nitrogen treatments on the activities of antioxidant enzymes (**A**–**H**); and sulfur metabolism related enzymes (**I**,**J**) in *O. sativa* roots. Bars represent means ± SD (*n* = 3). Different letters above the bars represent a significant difference at *p* < 0.05.

Nitrogen deficiency significantly lowered the activities of root APX (Figure 6A; decreased by 69.9%), root GuPX (Figure 6B; decreased by 69.9%), root SOD (Figure 6E; decreased by 84.8%), and root GlPX (Figure 6F; decreased by 48.1%) in *O. sativa* seedlings, whereas it increased the activities of root CAT (Figure 6C; increased by 318%), root DHAR (Figure 6D; increased by 25.2%), root GR (Figure 6G; increased by 62.8%), and root GST (Figure 6I; increased by 72.7%). Compared to the control, low N and N deficiency decreased the activity of root MDHAR (Figure 6H), but they did not change the activity of root OSALT in *O. sativa* seedlings (Figure 6J). Furthermore, no significant difference in these enzymes was observed between low-N and control root samples after three weeks of treatment (Figure 6). 

### 2.5. Effects of N Deficiency on the Contents of Antioxidant Compounds, MDA, and the Production of H_2_O_2_ in Rice Seedlings

**Figure 7:**
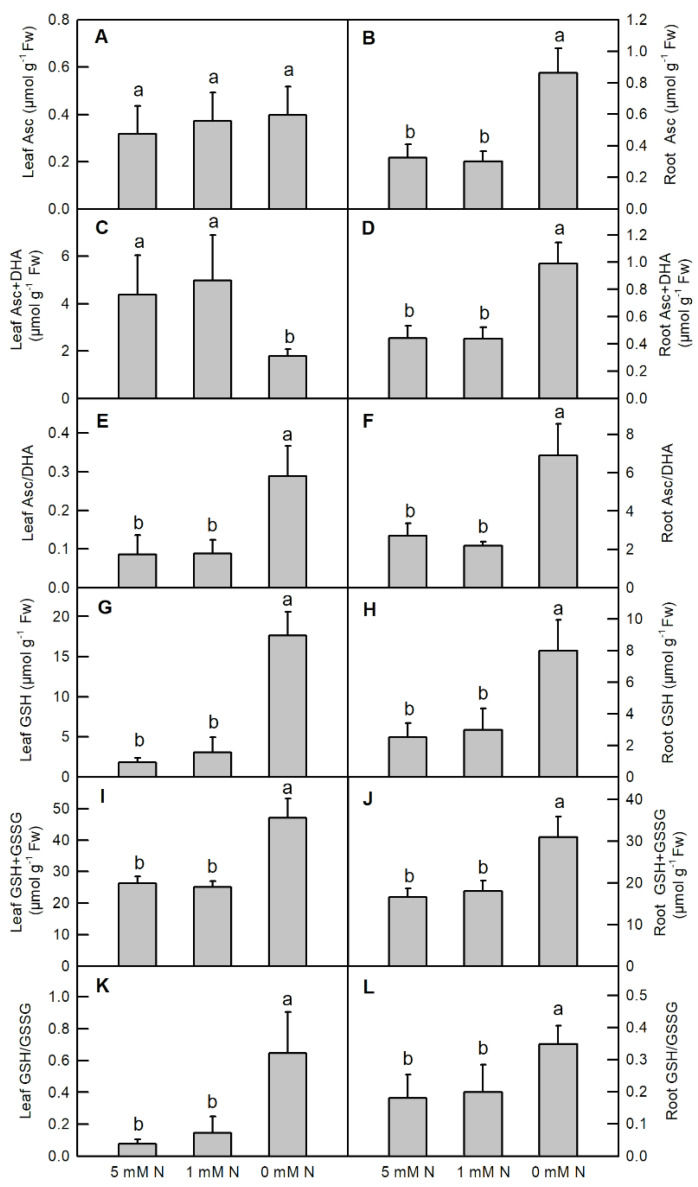
Effects of different nitrogen treatments on the contents of antioxidant compounds in the leaves (**A**,**C**,**E**,**G**,**I**,**K**); and roots (**B**,**D**,**F**,**H**,**J**,**L**) of rice. Bars represent means ± SD (*n* = 3). Different letters above the bars represent a significant difference at *p* < 0.05.

Nitrogen deficiency substantially increased the contents of root Asc (increased by 166%), root Asc+DHA (increased by 124%), leaf GSH (increased by 863%), root GSH (increased by 218%), leaf GSH+GSSG (increased by 79.3%), root GSH+GSSG (increased by 86.6%), and the ratios of leaf Asc/DHA, root Asc/DHA, leaf GSH/GSSG, and root GSH/GSSG in *O. sativa* (Figure 7B,D–L), but decreased the content of leaf Asc+DHA (Figure 7C). No significant difference was observed in the content of leaf Asc in *O. sativa* under different N supplies (Figure 7A). 

**Figure 8:**
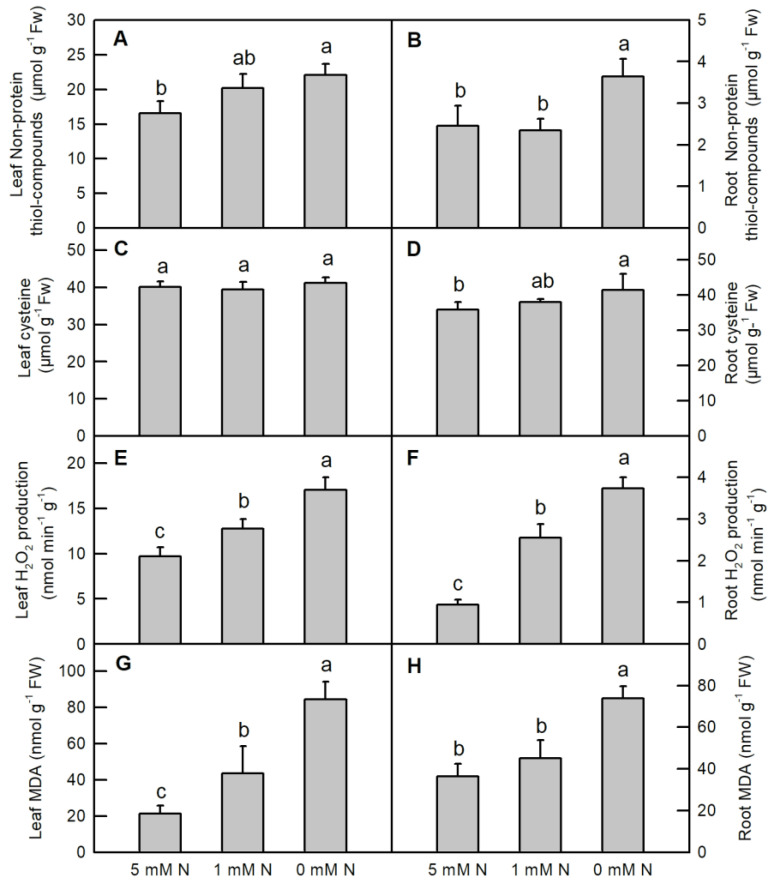
Effects of different nitrogen treatments on the contents of non-protein thiol compounds (**A**,**B**); cysteine (**C**,**D**); MDA (**E**,**F**); and the production of H_2_O_2_ (**G**,**H**) in the leaves and roots of rice. Bars represent means ± SD (*n* = 3). Different letters above the bars represent a significant difference at *p* < 0.05.

Compared to the 5 mM N treatment, N deficiency significantly raised the contents of leaf non-protein thiol compounds (NPTs) (Figure 8A), root NPTs (Figure 8B) and root cysteine (Figure 8D) in *O. sativa*. No significant difference in leaf NPTs (Figure 8A), root NPTs (Figure 8B) and root cysteine (Figure 8D) was observed between the 5 mM N and 1 mM N treatments. Different N levels did not change the content of leaf cysteine (Figure 8C). Reducing N levels gradually increased the production of H_2_O_2_ in the leaf and root, as well as the content of MDA (Figure 8E–G). N deficiency significantly increased root MDA when compared to both the control and low-N samples (Figure 8H).

### 2.6. Effects of N Deficiency on the Gene Expression Levels of Antioxidant Related Enzymes in Rice Seedlings

**Figure 9:**
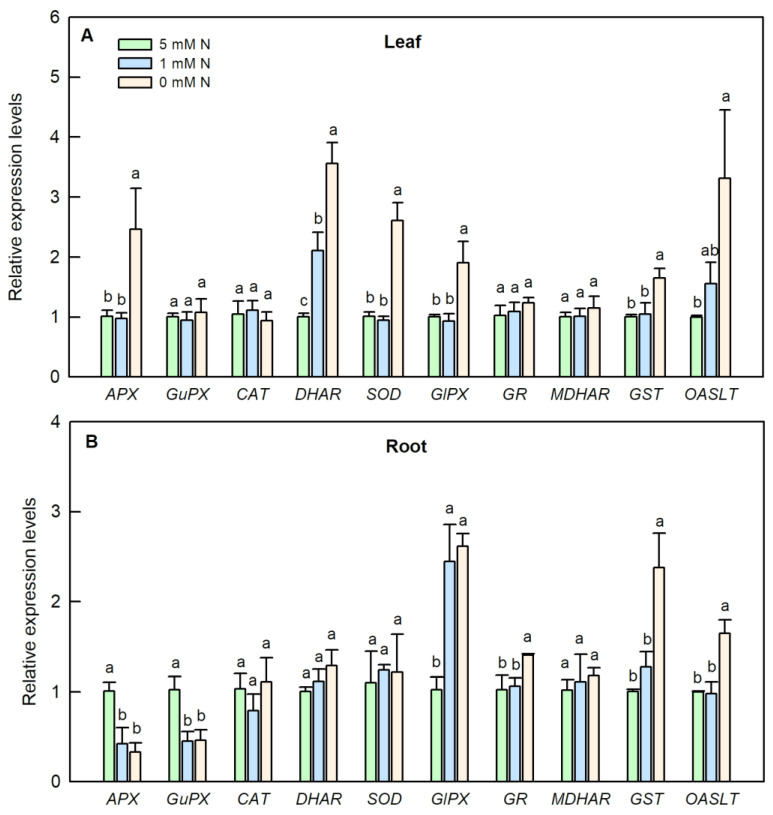
Effects of different nitrogen treatments on the gene expression patterns of antioxidant related enzymes in the leaves (**A**); and roots (**B**) of rice. Bars represent means ± SD (*n* = 3). Different letters above the bars of each gene represent a significant difference at *p* < 0.05.

Nitrogen deficiency apparently enhanced the gene expression levels of *APX*, *DHAR*, *SOD*, *GlPX*, *GST* and *OASTL*, whereas it did not change the expression levels of *GuPX*, *CAT*, *GR*, and *MDHAR* in *O. sativa* leaves (Figure 9A). Except for increasing the expression level of *DHAR*, the low-N treatment did not change the expression levels of the other abovementioned genes in *O. sativa* leaves when compared to the controls (Figure 9A). In *O. sativa* roots, both low N and N deficiency decreased the expression levels of *APX* and *GuPX* but increased the expression level of *GlPX*. Only N deficiency significantly enhanced the expression levels of *GR*, *GST* and *OASTL*, when compared to those of the controls (Figure 9B). There was no significant difference observed in the expression levels of *CAT*, *DHAR*, *SOD* and *MDHAR* among the three N treatments (Figure 9B).

## 3. Discussion

Nitrogen fertilizers are one of the most common fertilizers nowadays; they influence the genetic enhancement of grain yield and play a prominent role in the increment of grain production worldwide [32]. Therefore, understanding the physiological mechanisms underlying plant N nutrition could help to improve crop NUE, as maintaining crop productivity has multiple economic and environmental benefits. Our previous study indicated that low N and N deficiency disturbed the normal metabolism of organic acids and might reduce the carbon skeletons for amino acids assimilation in rice leaves [1]. Decreasing N supply significantly reduced the activities of enzymes related to N assimilation and amino acids metabolism, lowered the contents of total free amino acids (TFAAs) and soluble proteins, eventually hindering the growth of shoots and roots in *O. sativa* seedlings [1]. Here, we showed that decreasing N supply affected the normal growth of rice seedlings, reducing CO_2_ assimilation, stomatal conductance, chlorophyll *a*, chlorophyll *b*, and the ratio of Chl *a*/Chl *b*, but increasing the intercellular CO_2_ concentration (Figure 1 and Figure 2). The N deficiency induced decreased photosynthesis; related pigments were also reported in wheat [7], tea [14], sorghum [8], rice (cultivar “Zhendao 11” and “Weiyou916”) [2,9], sweet potato [12], sunflower [13], maize [10,11], *Citrus* [15], grape [33], and so on. Moreover, a decreased Chl *a*/Chl *b*, as an indicator of stress response in plants, was also found in N-deficient sunflower [13], copper-stress *Citrus* [34], and rice seedlings under aluminum treatment and P deficiency [35], indicating that like other abiotic stresses, N deficiency could disrupt the homeostasis of pigment metabolism and thereby lower the photosynthesis rate in *O. sativa* seedlings. Therefore, in order to confirm whether N deficiency impairs the photosynthetic electron transfer efficiency in the thylakoid membrane, we measured the chlorophyll *a* transient fluorescence and conducted the JIP test [36,37,38]. 

Our results showed that both low N and N deficiency displayed a raised F_o_, but a descending maximum of the IP_phase_ (Figure 3A,B and Figure 4A), implying that a photo-inhibitory damage to PS II or reaction center (RC) and the electron transfer block at the acceptor side of PSI occurred in *O. sativa* leaves under N-limiting conditions [37,39,40,41]. F_v_/F_m_ represents the maximum quantum yield of primary photochemistry. The decreased F_v_/F_m_ was only observed in N-deficient *O. sativa* leaves, which was caused by decreased F_m_ and increased F_o_ (Figure 4A–C). This result indicates that the photosynthetic capacity and stability of PSII are lower in *O. sativa* leaves under N-deficient than those under N -supplying conditions [42]. The K-step, J-step and I-step are correlated with the uncoupling of the oxygen-evolving complex (OEC), accumulation of reduced Q_A_ and inhibition of the final reduction of end acceptors, respectively [43]. The positive K-step, J-step and I-step, and the higher value of V_j_ and V_i_, observed in low-N and N-deficient *O. sativa* leaves indicated that N-limiting conditions could damage the OEC, and impair the whole photosynthetic electron transport chain (PETC) from the PSII donor side up to the reduction in the end acceptor side of PSI, affecting the energy migration properties within the PSs (Figure 3C,D and Figure 4D,E) [15,37,44]. The positive L-step appeared in the relative variable fluorescence between Fo to F_300μs_ (W_k_ and ΔW_k_ when compared to the control ones), demonstrating that the energy transmission between the independent PSII units is impaired or the PSII units are less grouped [36]. Furthermore, we selected some parameters based on the cross-section (CS_o_) at t = 0, which showed that leaf samples under low-N or N-deficient conditions absorbed more photo-radiation (ABS/CS_o_; Figure 4F), but these samples dissipated more energy (DI_o_/CS_o_; Figure 4G) and used less energy in electron transport (ET_o_/CS_o_; Figure 4H) or caused a reduction in the end acceptor side of PSI (Re_o_/CS_o_; Figure 4I), eventually reducing the total performance index of PSs (PI_total_; Figure 4J). The observed disruption at the donor side of PSII to the end acceptor side of PSI was also reported in N-deficient and copper-stress *Citrus* plants [15,34], phosphorus-deficient tea plants [38], N-deficient tomato and maize [6], and N-deficient wheat [7]. 

Abiotic stresses, including N deficiency, lower light energy utilization efficiency and excess photon energy, inevitably enhancing the production of ROS in the plants. As indicated in the current study, both low N and N deficiency enhanced the production of H_2_O_2_ in *O. sativa* leaves and roots (Figure 8E,F). Therefore, as a responsive strategy, the metabolism of antioxidant compounds and the activities of related enzymes are enhanced in response to adverse conditions. Here, we showed that the activities of ROS-detoxified related enzymes, such as leaf APX, GuPX, DHAR, SOD, GlPX, GR, sulfur-containing compounds, and metabolism-related enzymes, such as GST and OASTL, were enhanced by N deficiency in *O. sativa* leaves (Figure 5). Accordingly, the ratio of leaf Asc/DHA and leaf GSH/GSSG, and the contents of leaf GSH, total glutathione (GSH + GSSG) and non-protein thiol compounds were also increased by N deficiency in *O. sativa* leaves (Figure 7E,G,I,K and Figure 8A). The higher ratio of leaf Asc/DHA, leaf GSH/GSSG, and the higher contents of leaf GSH, total glutathione (GSH + GSSG) and non-protein thiol compounds might play positive roles in increasing the activities of leaf APX, DHAR, GlPX, GR, and GST, respectively, in *O. sativa* under N-deficient conditions. 

Although the activity of leaf OASTL was increased by N deficiency, the content of its product, cysteine, was not altered by N deficiency (Figure 5J and Figure 8C). This inconsistency might be due to the consumption of cysteine to synthesize proteins and GSH or due to it acting as a sulfur donor for the biosynthesis of methionine (Met) and some other secondary compounds such as *S*-adenosylmethionine and S-methylmethionine [45,46]. In contrast, except for increasing the activities of CAT, DHAR, GR, GST and OASTL, N deficiency decreased the activities of the other antioxidant enzymes mentioned above in *O. sativa* roots (Figure 6). Compared to the leaf, except for increasing the contents of GSH, GSH + GSSG, non-protein thiol compounds and the ratio of GSH/GSSG, N deficiency also increased the contents of Asc, Asc+DHA and cysteine, and the ratio of Asc/DHA in *O. sativa* roots (Figure 7 and Figure 8B,D). The increased ratios of Asc/DHA and GSH/GSSG in both leave and roots indicates that *O. sativa* has enhanced its antioxidant system to cope with oxidative stress induced by N deficiency both in the leaves and roots (Figure 7E,F,K,L) [47]. Interestingly, we found that the activity of CAT was decreased by N deficiency, along with the enhanced activity of APX and increased production of H_2_O_2_ in N-deficient *O. sativa* leaves, which might be due to the lower affinity of CAT to H_2_O_2_ (Km = 40 mM) than to APX [48]. Similar results were also reported in low-ammonium cultured *O. sativa* [49], and low-N cultured *Solanum lycopersicum* [18]. However, the low N-induced decreased activity of CAT needs to be further investigated. We proposed that the enhanced activities of CAT, DHAR, GR, the ASC–GSH cycle and the metabolism of sulfur-containing compounds might play protective roles in *O. sativa* roots under N-limiting conditions. Enhancing the capacity of the antioxidant enzymes and raising the contents of non-enzymatic antioxidants, such as reduced Asc, GSH, phenolic compounds, flavonoids, and sulfur-containing compounds, etc., is the main adaptive response to oxidative stress in plants [50]. The increased activities of antioxidant enzymes and contents of ascorbic acid and/or glutathione have also been reported in phosphorus-deficient *C. sinensis* [26], salinity-stress *Malus domestica* [51], and N-deficient *Nicotiana tabaccum* [52], *O. sativa* [17,21,22], *Cucumis sativus* [23], and *Matricaria chamomilla* [25]. In order to investigate whether the activities of these enzymes were transcriptionally regulated by their corresponding genes, we carried out the transcription profile of these enzymes related to the antioxidant system and sulfur metabolism using qRT-PCR assay. Our results showed that the expression patterns of most of the genes (70%) except for GuPX, CAT and GR, were consistent with the dynamics of the enzymes related to the antioxidant system and sulfur metabolism under N-limiting conditions in *O. sativa* leaves (Figure 9A). However, the expression patterns of only 60% of these genes coincided with the dynamics of the enzymes under N-limiting conditions in *O. sativa* roots (Figure 9B). The inconsistency of some enzymes between the gene expression level and enzyme activity might be attributed to translational regulations, post-translational modifications, and the protein modification or degradation of each enzyme [53]. These results indicate that the activities of most of these enzymes are controlled by the transcriptional regulation of corresponding genes in *O. sativa* under N-limiting conditions.

## 4. Materials and Methods

### 4.1. Plant Material and Treatment

Plant culture and treatment were the same as the methods described in our previous study [1]. Briefly, uniform seeds of the rice variety “Huanghuazhan” (*Oryza sativa* L. ssp. Indica) were sown in a plastic tray containing natural paddy soil. The seeds were kept moist in a greenhouse under natural light and temperatures. When the height of the rice seedlings were about 15 cm, the seedlings were transplanted to the cultivation bucket containing Hoagland nutrient solutions with the following three different N concentrations: 5 mM NH_4_NO_3_ (control); 1 mM NH_4_NO_3_ (low N); and 0 mM NH_4_NO_3_ (N deficiency). All the rice seedlings were cultivated in a light incubator under a constant temperature of 28 °C. The nutrient solutions were refreshed every two days. The Hoagland nutrient solution contained the following macro-elements except for N (in mM): KH_2_PO_4_, 1 mM; MgSO_4_, 2 mM; and microelements (in μM): H_3_BO_3_, 5 μM; CuSO_4_, 0.5 μM; MnCl_2_, 2 μM; (NH_4_)_6_Mo_7_O_24_, 0.065 μM; ZnSO_4_, 2 μM; FeSO_4_-EDTA, 20 μM; and Na_2_SiO_3_, 0.1 μM. Three weeks later, rice seedlings were separated into shoots and roots and wrapped with aluminum foil. All the samples were immediately frozen in liquid nitrogen and stored at −80 °C until assayed. The experimental conditions for all the seedlings were the same except for the N levels in the nutrient solution.

### 4.2. Measurement of Leaf Gas Exchange, Chlorophyll a Transient Fluorescence (OJIP) and the Contents of Chlorophyll in Rice Leaves

After the end of the different N treatments, leaf gas exchange was monitored with a CIARS-II portable photosynthesis system (PP systems, Amesbury, MA, USA) between 10:00 am and 11:30 am on a clear day. The CO_2_ concentration was supplied by a CO_2_ cylinder and controlled at ~380 µmol mol^–1^. The controlled light intensity was 1000 µmol m^−2^ s^−1^. During the measurements, the leaf temperature and vapor pressure deficit (VPD) were 29.6 ± 0.92 °C and 1.6 ± 0.12 kPa, respectively. The chlorophyll *a* transient fluorescence was measured using the portable Handy PEA (Hansatech Instruments, Norfolk, UK) after dark adaptation for 90 minutes. Relative OJIP transient parameters were calculated according to the JIP test [37]. Leaf chlorophylls were extracted with 80% (*v*/*v*) acetone in the dark and the absorbance of the extraction was measured at 470 nm, 645 nm, and 663 nm, respectively [54].

### 4.3. Extraction and Measurement of Antioxidant Metabolites and Malondialdehyde (MDA)

Reduced ascorbate (Asc) and dehydroascorbate (DHA) were extracted with ice-cold 6% (*v*/*v*) HClO_4_ and measured according to the method reported by Longan [37]. Reduced glutathione (GSH) and oxidized glutathione (GSSG) were extracted with ice-cold 5% (*w*/*v*) trichloroacetic acid (TCA) and determined according to the protocol described by Chen and Cheng [33]. MDA in the leaf and root of *O. sativa* was extracted with 80% (*v*/*v*) ethanol and represented as the thiobarbituric acid reactive substances (TBARS) [55]. 

### 4.4. Extraction and Measurement of Non-Protein Thiol Compounds (NPTs)

The content of NPTs was assayed according to the method described by Dixit et al. [56]. Briefly, about 50 mg of leaf or root samples were ground with 1.5 mL extraction buffer (pH = 8.0) containing 150 mM Tris–HCl and 5.5 mM EDTA. After being centrifuged at 12,000× *g* for 10 minutes at 4 °C, the supernatant was used to measure the content of NPTs in a 1 mL reaction solution containing 150 mM Tris–HCl (pH = 8.0), 5.5 mM EDTA, 1.2 mM DTNB. The reaction solution was allowed to stand at room temperature for 10 minutes and then centrifuged at 5000× *g* for 10 minutes at 4 °C. The obtained supernatant was used to monitor the absorbance at 412 nm. The contents of cysteine were extracted and measured according to the method previously described by Zhang et al. [57].

### 4.5. Extraction and Measurement of Antioxidant Enzymes 

SOD, CAT, APX, GuPX, GlPX, DHAR, MDHAR, GR, GST and OASTL were extracted with buffer solution containing KH_2_PO_4_-KOH (50 mM, pH = 7.5), EDTA (0.1 mM), Triton X-100 (0.3%, *w*/*v*), and insoluble polyvinylpolypyrrolidone (4%, *w*/*v*) [55]. 

The activity of SOD was measured according to a previously described method [58]. The activities of APX, CAT, GuPX, DHAR, MDHAR and GR were assayed according to Chen et al. [59]. The activities of GlPX and GST were assayed according to Cai et al. [55]. The activity of OASTL was assayed by the method described by Warrilow and Hawkesford [60]. 

### 4.6. Quantitative Real-Time PCR (qRT-PCR) Analysis

Total RNA was extracted from *O. sativa* leaves and roots using a RNAprep Pure Plant Plus Kit (Tiangen, Beijing, China). After the integrity and concentration of total RNA were assessed, total RNA was reversely transcribed into single-strand cDNA using a MMLV transcriptase. The gene special primer pairs were designed by PrimerPremier software (Version 5.0) using the mRNA sequences of *O. sativa* genome data deposited in NCBI (https://www.ncbi.nlm.nih.gov/assembly/GCF_001433935.1, accessed on 15 July 2024) (Table S1). qRT-qPCR was carried out using iQ SYBR green super-mix kit with a CFX96 Real-time System (Bio–Rad, Hercules, CA, USA). The PCR procedure was as follows: 95 °C, 2 min; 40 cycles of 95 °C, 10 s, 60 °C, 10 s, 72 °C, 30 s. The relative expression level of each gene was calculated by the 2^−ΔΔCt^ method [61]. *Ubiquitin* (accession number: LOC107277317) was used as the internal control gene and the control samples were set as the reference samples. Three biological replicates per sample were analyzed for the expression levels.

### 4.7. Experiment Design and Statistical Analysis

Three culture pots were set for each N treatment, and each culture pot contained 24 *O. sativa* seedlings. The experimental data were processed by Microsoft Excel 2010, analyzed by SPSS 19.0, and visualized by Sigmaplot 10.0. Differences between means were separated by a least significant difference (LSD) test at *p*-value < 0.05.

## 5. Conclusions

Decreasing the N supply affected the normal growth of *O. sativa* seedlings, reducing CO_2_ assimilation, stomatal conductance, the contents of chlorophylls, and the ratio of Chl *a*/Chl *b*, but increasing the intercellular CO_2_ concentration. The JIP test results indicated that N deficiency increased F_o_ but decreased the F_v_/F_m_ and the maximum of the IP_phase_, implying that N-limiting conditions impaired the whole photo electron transport chain from the donor side of PSII to the end acceptor side of PSI in *O. sativa*. N deficiency enhanced the activities of antioxidant enzymes, such as APX, GuPX, DHAR, SOD, GuPX, GR, GST and OASTL, and the contents of antioxidant compounds, including GSH, GSH + GSSG, and non-protein thiol compounds in *O. sativa* leaves. In contrast, the increased activities of CAT, DHAR, GR, GST and OASTL, the enhanced ASC–GSH cycle, and the content of sulfur-containing compounds might provide protective roles against oxidative stress in *O. sativa* roots under N-limiting conditions. When the results of the qRT-PCR analysis and the measurement of enzyme activity were combined, it was found that the number of enzymes was larger in *O. sativa* leaves than in roots; however, the dynamics of enzyme activity were consistent with their gene expression patterns under N–limiting conditions.

## Data Availability

Data will be made available on request.

## Supplementary Materials

The following supporting information can be downloaded at: https://www.mdpi.com/article/10.3390/ijms251910409/s1.

## References

[1] Chen L.H., Cheng Z.X., Xu M., Yang Z.J., Yang L.T. (2022). Effects of nitrogen deficiency on the metabolism of organic acids and amino acids in *Oryza sativa*. Plants.

[2] Shao C.H., Qiu C.F., Qian Y.F., Liu G.R. (2020). Nitrate deficiency decreased photosynthesis and oxidation-reduction processes, but increased cellular transport, lignin biosynthesis and flavonoid metabolism revealed by RNA-Seq in *Oryza sativa* leaves. PLoS ONE.

[3] Peng J., Feng Y., Wang X., Li J., Xu G., Phonenasay S., Luo Q., Han Z., Lu W. (2021). Effects of nitrogen application rate on the photosynthetic pigment, leaf fluorescence characteristics, and yield of indica hybrid rice and their interrelations. Sci. Rep..

[4] Lee S. (2021). Recent advances on nitrogen use efficiency in rice. Agronomy.

[5] Shrestha S., Brueck H., Asch F. (2012). Chlorophyll index, photochemical reflectance index and chlorophyll fluorescence measurements of rice leaves supplied with different N levels. J. Photochem. Photobiol. B Biol..

[6] Kalaji H.M., Oukarroum A., Alexandrov V., Kouzmanova M., Brestic M., Zivcak M., Samborska I.A., Cetner M.D., Allakhverdiev S.I., Goltsev V. (2014). Identification of nutrient deficiency in maize and tomato plants by *in vivo* chlorophyll *a* fluorescence measurements. Plant Physiol. Biochem..

[7] Kang J., Chu Y., Ma G., Zhang Y., Zhang X., Wang M., Lu H., Wang L., Kang G., Ma D. (2023). Physiological mechanisms underlying reduced photosynthesis in wheat leaves grown in the field under conditions of nitrogen and water deficiency. Crop J..

[8] Zhao D., Reddy K.R., Kakani V.G., Reddy V.R. (2005). Nitrogen deficiency effects on plant growth, leaf photosynthesis, and hyperspectral reflectance properties of sorghum. Eur. J. Agron..

[9] Gao L., Lu Z., Ding L., Xie K., Wang M., Ling N., Guo S. (2020). Anatomically induced changes in rice leaf mesophyll conductance explain the variation in photosynthetic nitrogen use efficiency under contrasting nitrogen supply. BMC Plant Biol..

[10] Ning P., Yang L., Li C., Fritschi F.B. (2018). Post-silking carbon partitioning under nitrogen deficiency revealed sink limitation of grain yield in maize. J. Exp. Bot..

[11] Biswas D.K., Jiang G.M., Xu H., Wang K.J., Ding L., Li L.F., Li Y.H. (2005). Effects of nitrogen deficiency on photosynthetic traits of maize hybrids released in different years. Ann. Bot..

[12] Meng W., Aijun Z., Hongmin L., Zhonghou T., Xiaoguang C. (2015). Growth and physiological response to nitrogen deficiency and re-supply in leaf-vegetable sweetpotato (*Ipomoea batatas* Lam). Hortscience.

[13] Agüera E., Cabello P., De La Haba P. (2010). Induction of leaf senescence by low nitrogen nutrition in sunflower (*Helianthus annuus*) plants. Physiol. Plant..

[14] Lin Z.H., Zhong Q.S., Chen C.S., Ruan Q.C., Chen Z.H., You X.M. (2016). Carbon dioxide assimilation and photosynthetic electron transport of tea leaves under nitrogen deficiency. Bot. Stud..

[15] Huang W.T., Xie Y.Z., Chen X.F., Zhang J., Chen H.H., Ye X., Guo J., Yang L.T., Chen L.S. (2021). Growth, mineral nutrients, photosynthesis and related physiological parameters of *Citrus* in response to nitrogen deficiency. Agronomy.

[16] Tantray A.Y., Bashir S.S., Ahmad A. (2020). Low nitrogen stress regulates chlorophyll fluorescence in coordination with photosynthesis and Rubisco efficiency of rice. Physiol. Mol. Biol. Plants.

[17] Huang Z.A., Jiang D.A., Yang Y., Sun J.W., Jin S.H. (2004). Effects of nitrogen deficiency on gas exchange, chlorophyll fluorescence, and antioxidant enzymes in leaves of rice plants. Photosynthetica.

[18] Machado J., Vasconcelos M.W., Soares C., Fidalgo F., Heuvelink E., Carvalho S.M.P. (2023). Enzymatic and non-enzymatic antioxidant responses of young tomato plants (cv. Micro-tom) to single and combined mild nitrogen and water deficit: Not the sum of the parts. Antioxidants.

[19] Gill S.S., Tuteja N. (2010). Reactive oxygen species and antioxidant machinery in abiotic stress tolerance in crop plants. Plant Physiol. Biochem.

[20] Foyer C.H., Shigeoka S. (2011). Understanding oxidative stress and antioxidant functions to enhance photosynthesis. Plant Physiol..

[21] Liang Z., Bao A., Li H., Cai H. (2015). The effect of nitrogen level on rice growth, carbon-nitrogen metabolism and gene expression. Biologia.

[22] Lin Y.L., Chao Y.Y., Huang W.D., Kao C.H. (2011). Effect of nitrogen deficiency on antioxidant status and Cd toxicity in rice seedlings. Plant Growth Regul..

[23] Zhang X., Yu H.J., Zhang X.M., Yang X.Y., Zhao W.C., Li Q., Jiang W.J. (2016). Effect of nitrogen deficiency on ascorbic acid biosynthesis and recycling pathway in cucumber seedlings. Plant Physiol. Biochem..

[24] Kumar Tewari R., Kumar P., Tewari N., Srivastava S., Sharma P.N. (2004). Macronutrient deficiencies and differential antioxidant responses-influence on the activity and expression of superoxide dismutase in maize. Plant Sci..

[25] Kováčik J., Bačkor M. (2007). Changes of phenolic metabolism and oxidative status in nitrogen-deficient Matricaria chamomilla plants. Plant Soil.

[26] Lin Z.H., Chen L.S., Chen R.B., Zhang F.Z. (2012). Antioxidant system of tea (*Camellia sinensis*) leaves in response to phosphorus supply. Acta Physiol. Plant.

[27] Narayan O.P., Kumar P., Yadav B., Dua M., Johri A.K. (2023). Sulfur nutrition and its role in plant growth and development. Plant Signal. Behav..

[28] Cao Y., Ma C., Yu H., Tan Q., Dhankher O.P., White J.C., Xing B. (2023). The role of sulfur nutrition in plant response to metal(loid) stress: Facilitating biofortification and phytoremediation. J. Hazard. Mater..

[29] Aarabi F., Naake T., Fernie A.R., Hoefgen R. (2020). Coordinating sulfur pools under sulfate deprivation. Trends Plant Sci..

[30] Lin Z.H., Chen C.S., Zhong Q.S., Ruan Q.C., Chen Z.H., You X.M., Shan R.Y., Li X.L. (2021). The GC-TOF/MS-based metabolomic analysis reveals altered metabolic profiles in nitrogen-deficient leaves and roots of tea plants (*Camellia sinensis*). BMC Plant Biol..

[31] Huang W.T., Zheng Z.C., Hua D., Chen X.F., Zhang J., Chen H.H., Ye X., Guo J.X., Yang L.T., Chen L.S. (2022). Adaptive responses of carbon and nitrogen metabolisms to nitrogen-deficiency in *Citrus sinensis* seedlings. BMC Plant Biol..

[32] Jewel Z.A., Ali J., Mahender A., Hernandez J., Pang Y., Li Z. (2019). Identification of quantitative trait loci associated with nutrient use efficiency traits, using snp markers in an early backcross population of rice (*Oryza sativa* L.). Int. J. Mol. Sci..

[33] Chen L.S., Cheng L.L. (2003). Both xanthophyll cycle-dependent thermal dissipation and the antioxidant system are up-regulated in grape (*Vitis labrusca* L. *cv. Concord*) leaves in response to N limitation. J. Exp. Bot..

[34] Lu F., Hu P., Lin M., Ye X., Chen L., Huang Z. (2022). Photosynthetic characteristics and chloroplast ultrastructure responses of citrus leaves to copper toxicity induced by bordeaux mixture in greenhouse. Int. J. Mol. Sci..

[35] Guo T.R., Yao P.C., Zhang Z.D., Wang J.J., Wang M. (2012). Involvement of antioxidative defense system in rice seedlings exposed to aluminum toxicity and phosphorus deficiency. Rice Sci..

[36] Strasser R.J., Tsimilli-Michael M., Srivastava A., Papageorgiou G.C., Govindjee (2004). Analysis of the Chlorophyll a Fluorescence Transient. Chlorophyll a Fluorescence: A Signature of Photosynthesis.

[37] Yang L.T., Qi Y.P., Chen L.S., Sang W., Lin X.J., Wu Y.L., Yang C.J. (2012). Nitric oxide protects sour pummelo (*Citrus grandis*) seedlings against aluminum-induced inhibition of growth and photosynthesis. Environ. Exp. Bot..

[38] Lin Z.H., Chen L.S., Chen R.B., Zhang F.Z., Jiang H.X., Tang N. (2009). CO_2_ assimilation, ribulose-1,5-bisphosphate carboxylase/oxygenase, carbohydrates and photosynthetic electron transport probed by the JIP-test, of tea leaves in response to phosphorus supply. BMC Plant Biol..

[39] Šetlík I., Allakhverdiev S.I., Nedbal L., Šetlíková E., Klimov V.V. (1990). Three types of Photosystem II photoinactivation. Photosynth. Res..

[40] Franklin L.A., Levavasseur G., Osmond C.B., Henley W.J., Ramus J. (1992). Two components of onset and recovery during photoinhibition of Ulva rotundata. Planta.

[41] Schansker G., Tóth S.Z., Strasser R.J. (2005). Methylviologen and dibromothymoquinone treatments of pea leaves reveal the role of photosystem I in the Chl *a* fluorescence rise OJIP. Biochim. Biophys. Acta.

[42] Chen J.H., Chen S.T., He N.Y., Wang Q.L., Zhao Y., Gao W., Guo F.Q. (2020). Nuclear-encoded synthesis of the D1 subunit of photosystem II increases photosynthetic efficiency and crop yield. Nat. Plants.

[43] Smit M.F., van Heerden P.D.R., Pienaar J.J., Weissflog L., Strasser R.J., Krüger G.H.J. (2009). Effect of trifluoroacetate, a persistent degradation product of fluorinated hydrocarbons, on *Phaseolus vulgaris* and *Zea mays*. Plant Physiol. Biochem..

[44] Srivastava A., Guissé B., Greppin H., Strasser R.J. (1997). Regulation of antenna structure and electron transport in Photosystem II of *Pisum sativum* under elevated temperature probed by the fast polyphasic chlorophyll a fluorescence transient: OKJIP. Biochim. Biophys. Acta (BBA) Bioenerg..

[45] Hesse H., Nikiforova V., Gakière B., Hoefgen R. (2004). Molecular analysis and control of cysteine biosynthesis: Integration of nitrogen and sulphur metabolism. J. Exp. Bot..

[46] Zenzen I., Cassol D., Westhoff P., Kopriva S., Ristova D. (2024). Transcriptional and metabolic profiling of sulfur starvation response in two monocots. BMC Plant Biol..

[47] Siddiqui M.H., Alamri S., Al-Khaishany M.Y., Khan M.N., Al-Amri A., Ali H.M., Alaraidh I.A., Alsahli A.A. (2019). Exogenous melatonin counteracts nacl-induced damage by regulating the antioxidant system, proline and carbohydrates metabolism in tomato seedlings. Int. J. Mol. Sci..

[48] Zhang Z., Xu Y., Xie Z., Li X., He Z.H., Peng X.X. (2016). Association–dissociation of glycolate oxidase with catalase in rice: A potential switch to modulate intracellular H_2_O_2_ Levels. Mol. Plant.

[49] Wu Z., Jiang Q., Yan T., Zhang X., Xu S., Shi H., Deng T.-H.-B., Li F., Du Y., Du R. (2020). Ammonium nutrition mitigates cadmium toxicity in rice (*Oryza sativa* L.) through improving antioxidase system and the glutathione-ascorbate cycle efficiency. Ecotoxicol. Environ. Saf..

[50] Fujita M., Hasanuzzaman M. (2022). Approaches to enhancing antioxidant defense in plants. Antioxidants.

[51] Sun T., Pei T., Yang L., Zhang Z., Li M., Liu Y., Ma F., Liu C. (2021). Exogenous application of xanthine and uric acid and nucleobase-ascorbate transporter MdNAT7 expression regulate salinity tolerance in apple. BMC Plant Biol..

[52] Rubio-Wilhelmi M.M., Sanchez-Rodriguez E., Rosales M.A., Begoña B., Rios J.J., Romero L., Blumwald E., Ruiz J.M. (2011). Effect of cytokinins on oxidative stress in tobacco plants under nitrogen deficiency. Environ. Exp. Bot..

[53] Pradet-Balade B., Boulmé F., Beug H., Müllner E.W., Garcia-Sanz J.A. (2001). Translation control: Bridging the gap between genomics and proteomics?. Trends Biochem. Sci..

[54] Lichtenthaler H.K., Wellburn A.R. (1983). Determination of total carotenoids and chlorophyll a and b of leaf extracts in different solvents. Biochem. Soc. Trans..

[55] Cai Y.T., Zhang H., Qi Y.P., Ye X., Huang Z.R., Guo J.X., Chen L.S., Yang L.T. (2019). Responses of reactive oxygen species and methylglyoxal metabolisms to magnesium-deficiency differ greatly among the roots, upper and lower leaves of *Citrus sinensis*. BMC Plant Biol..

[56] Dixit G., Singh A.P., Kumar A., Singh P.K., Kumar S., Dwivedi S., Trivedi P.K., Pandey V., Norton G.J., Dhankher O.P. (2015). Sulfur mediated reduction of arsenic toxicity involves efficient thiol metabolism and the antioxidant defense system in rice. J. Hazard. Mater..

[57] Zhang X., Kang J., Pang H., Niu L., Lv J. (2018). Sulfur mediated improved thiol metabolism, antioxidant enzymes system and reduced chromium accumulation in oilseed rape (*Brassica napus* L.) shoots. Environ. Sci. Pollut. Res..

[58] Giannopolitis C.N., Ries S.K. (1977). Superoxide dismutases: I. Occurrence in higher plants. Plant Physiol..

[59] Chen L.S., Li P., Cheng L. (2008). Effects of high temperature coupled with high light on the balance between photooxidation and photoprotection in the sun-exposed peel of apple. Planta.

[60] Warrilow A.G., Hawkesford M.J. (2000). Cysteine synthase (O-acetylserine (thiol) lyase) substrate specificities classify the mitochondrial isoform as a cyanoalanine synthase. J. Exp. Bot..

[61] Livak K.J., Schmittgen T.D. (2001). Analysis of relative gene expression data using real-time quantitative PCR and the 2^−ΔΔCT^ method. Methods.

