# Supplementary material for: Effects of Nitrogen Deficiency on the Photosynthesis, Chlorophyll a Fluorescence, Antioxidant System, and Sulfur Compounds in Oryza sativa
Source: Int J Mol Sci. 2024 Sep 27;25(19):10409. doi: 10.3390/ijms251910409 (PMC11476759; doi:10.3390/ijms251910409)
Supplement: Supplementary file 1 [file ijms-25-10409-s001.zip › ijms-3208633-supplementary.pdf]

Table S1. Primer pairs of the antioxidant enzymes used for qRT-PCR analysis.

Gene ID	Description	Forward primer (5'-3')	Reverse primer (5'-3')
LOC4332474	L-ascorbate peroxidase 1(APX)	GATACCCACCATCTCCTACGCC	GGTTTCTTGTCCAAGGTCCCTC
LOC4327001	Guaiacol peroxidase 1 (GuPX)	CCGCCTCCACTTCCACGACT	CGGTTAGCTTGACGCTGTCGC
LOC4328073	Catalase (CAT)	ACGAAGACGACGACGACGAA	ATGACGGTGGAGAAGCGGAC
LOC4337622	Dehydro-ascorbate reductase 1 (DHAR)	TCTTGTCACCCCTCCTGAGTATG	GCTGAAATGTTCTGCCCGTTG
LOC4332846	[Cu-Zn] superoxide dismutase (SOD)	TTGGAAAGGGTGGGCACGAG	ACGATGCGGGCGACAGAATG
LOC4325768	Glutathione transferase (GST)	CCATCGGCTACCTCGACCTG	ATTATCCCACGCTTCATTTTGC
LOC4332876	Glutathione peroxidase (GlPX)	ATCTACCCGCTCCGCTCCTA	GGCTCACCTCCTTGCCGTTG
LOC4352839	O-acetylserine(thiol) lyase (OASTL)	GCCATCGGGTCTATCTCCTGC	AACGGCGTGTTCCCAATCAA
LOC4331112	Glutathione reductase (GR)	TGATTGAAGGGGCAGGCAGTT	CCGCCACCAAGGATTACAGC
LOC4330469	Monodehydroascorbate reductase (MDHAR)	TCTTCCCCAATCCAACCCAA	CGTTCATAAGGAGCAACCGTCTC
LOC107277317	Ubiquitin-60S ribosomal protein	AGGAGTCCACGCTCCACCTC	ATCATCGTTTCGTTCGCTTTTC
